# Observational Study of Trans-Septal Endocardial Left Ventricle Lead Implant for Effective Cardiac Resynchronization Therapy in Patients with Heart Failure and Challenging Coronary Sinus Anatomy

**DOI:** 10.3390/biomedicines12122693

**Published:** 2024-11-26

**Authors:** Arsalan Farhangee, Mark J. Davies, Katie Gaughan, Mihai Mesina, Ion Mîndrilă

**Affiliations:** 1 Department of Cardiology, Milton Keynes University Hospital, Milton Keynes MK6 5LD, UK; 2 Department of Cardiology, Plymouth NHS Trust Foundation, Derriford Hospital, Plymouth PL6 8DH, UK; 3 Department of Cardiology, United Lincolnshire NHS Trust, Lincoln County Hospital, Lincoln LN2 5QY, UK; 4 Department of Cardiology, Oxford University Hospital, John Radcliffe Hospital, Oxford OX3 9DU, UK; 5 Doctoral School, University of Medicine and Pharmacy of Craiova, 200349 Craiova, Romania

**Keywords:** cardiac resynchronization therapy, trans-septal LV lead, heart failure, quality of life

## Abstract

Background: When conventional trans-venous CS lead placement fails, trans-septal endocardial left ventricle lead placement is an alternative technique used to capture the left ventricle endocardially; however, its use is limited due to a lack of evidence, practice uptake, and clinical trials. Methods: In this single-center cohort study, we evaluated the efficiency of the procedure, post-procedural complication rate, rate of thromboembolic events, overall survival rate, and changes in the echocardiographic parameters, brain natriuretic peptide (BNP) level, and New York Heart Association (NYHA) class, both before and after TSLV lead implantation. Results: The TSLV lead implant is safe and improves EF, LVEDV, LVESV, and LVIDd. It significantly reduces the NTproBNP levels and the NYHA class; however, the rate of stroke incidence remains high, at 9%. Conclusions: We demonstrated a high success rate of trans-septal left ventricular endocardial lead implantation, LV reverse remodeling was noted, and patients had a favorable clinical response; however, there was an increased risk of systemic embolization after the trans-septal LV lead implant.

## 1. Introduction

Several trials have been conducted to assess the role of cardiac resynchronization therapy (CRT) in managing heart failure, demonstrating its benefits. Combined with optimal medical treatment (OMT), CRT has been shown to improve the left ventricle’s structure, functional New York Heart Association (NYHA) class, and quality of life while reducing the risk of hospitalization due to heart failure [1,2,3,4]. Research on CRT is increasingly focused on special subgroups [5,6] and alternative techniques to biventricular pacing [7,8]. The conventional method of CRT implantation is implanting a left ventricle (LV) lead into a side branch of the coronary sinus tributary to pace the epicardial surface and capture the LV. This is safe and well tolerated with a high success rate [9]. The rate of failure in placing an LV lead has decreased over time. In a large study by Gamble et al., involving 29,503 patients, the overall rate of failed LV lead placement was 3.6%, including the inability to cannulate the coronary sinus (CS), an unsuitable target vein, and phrenic nerve stimulation. The study found that, beyond technical limitations, the reasons for failing to place a conventional LV lead were predominantly due to an adverse anatomy [10]. The extent of myocardial scarring and viability also play a major role that could lead to high capture thresholds or non-response to CRT and the irreversible correction of left bundle branch block (LBBB). Ypenburg et al. conducted a study among 51 patients post CRT implant for ischemic heart disease and left ventricle desynchrony. Photon emission-computed tomography (SPECT) with a technetium of 99 m tetrofosmin was performed to assess the scar tissue and viable myocardium of 867 segments, demonstrating a direct correlation of response to CRT based on segment viability [11]. These hypotheses have spurred the exploration of alternative techniques aimed at improving outcomes for patients who may not respond favorably to traditional CRT.

Trans-septal endocardial left ventricle lead placement is an alternative technique used to capture the left ventricle endocardially, but its use is limited due to a lack of evidence, practice uptake, and clinical trials. There has been a paucity of retrospective studies evaluating the risk of complications, the safety and efficacy of this procedure, and the extent of long-term complications. Geller et al. studied 44 patients who underwent trans-septal LV endocardial lead implantation; over a median follow-up of 29 months, despite an effective approach, this intervention was associated with a higher rate of thromboembolic cerebrovascular accidents (CVAs) of 7% [12]. In another study by van Gelder et al., trans-septal LV lead implantation was successful in nine out of ten patients, with a stable stimulation threshold and no thromboembolic events upon appropriate anticoagulation [13]. Current guidelines do not provide a definitive recommendation for the choice of anticoagulant, and, in clinical practice, various factors could influence it. Future randomized controlled trials and large cohort studies could provide insights into the optimal choice of long-term anticoagulation for these patients.

Neuhoff et al., in a case series following four patients who underwent trans-septal endocardial LV lead implant, demonstrated favorable LV reverse remodeling, with an improvement in functional status and no major post-implant complications [14]. Other alternative methods largely used in this group of patients include implanting a surgical epicardial LV lead. However, this is often deemed to constitute a high risk.

Leadless left ventricular stimulation with a wise-CRT system, conduction system pacing, and left bundle branch area pacing (LBBAP) are new emerging alternative techniques to trans-venous CRT; however, questions remain regarding several areas of leadless LBBAP or conduction system pacing relating to their efficacy and effective response [15,16,17,18].

Since affected patients are experiencing heart failure and are dependent on device therapy, alternative approaches are considered for those with a failed trans-venous LV lead implant on a case-by-case basis, following a personalized approach to treatment and the mitigation of heart failure [19].

In this observational study conducted in a single tertiary center, we evaluated the efficiency of the procedure, the post-procedural complication rate, the rate of thromboembolic events, and changes in echocardiographic parameters, brain natriuretic peptide (BNP) level, and New York Heart Association (NYHA) class.

## 2. Materials and Methods

### 2.1. Study Design

This retrospective study conducted in a single tertiary center encompassed a total of 14 (fourteen) patients with a failed CS left ventricle lead who had undergone a trans-septal endocardial lead implant.

The aim of this study was to investigate the safety and efficacy of trans-septal left ventricular endocardial lead implants. The analysis focused on changes in echocardiographic parameters such as left ventricle ejection fraction, left ventricle end-systolic volume, and left ventricle end-diastolic volume, comparing baseline to post-TSLV implant values, as well as changes in functional NYHA class, overall survival, and post-procedural complications.

The datasets for analysis were collected from Derriford Hospital, Plymouth Hospital, NHS trusts, and included information from a total of 14 patients who had undergone a cardiac resynchronization therapy upgrade using a trans-septal left ventricle endocardial lead implant between 2008 and 2021. Three patients were ineligible on the grounds of incomplete data and were excluded from this study. The decision to implant an endocardial LV lead was made by a multidisciplinary team, and the participants had experienced at least one failed attempt to implant a trans-venous LV lead.

The reasons behind the failed conventional trans-venous LV lead were the following: one case was due to persistent phrenic nerve stimulation; three cases were due to high pacing thresholds; one case was due to post-LV lead extraction; and six other cases were either due to a difficulty in cannulating the coronary sinus or the inability to identify a suitable CS tributary side branch.

All 11 interventions had been performed by the same operator and under general anesthesia. The same technique was used in all patients—the GooseNeck snare technique.

#### 2.1.1. Pacemaker Interrogation

Participants were followed up at 6 weeks after TSLV implantation and every 6 months afterwards. Optimization was performed to ensure effective CRT.

#### 2.1.2. Echocardiography

Parameters, including LVEF (assessed by Simpson’s biplane method), left ventricle end-systolic volume, left ventricle end-diastolic volume, and left ventricle internal diameter in diastole were analyzed from baseline and up to 12 months after TSLV implantation.

#### 2.1.3. Patient Characteristics

Patient characteristics, including NYHA functional classification, associated comorbidities, overall survival (OS), BNP/NTproBNP levels, and post-procedure-related complications were obtained from patients’ electronic and physical medical records.

#### 2.1.4. Patient Selection

We enrolled patients with TSLV lead implants with a guideline-based indication for CRT who had a failed LV lead implant due to failed coronary sinus (CS) cannulation, inability to canulate the CS tributary, phrenic nerve stimulation, or high pacing thresholds. These patients were referred for a TSLV lead implant because optimal medical therapy often failed to manage their heart failure with recurrent hospitalizations for decompensated heart failure. In most cases, they were in functional NYHA class III or IV, with worsening heart failure and echocardiographic features.

The main criteria for patients to be enrolled in the study were the availability of both pre- and post-TSLV lead implant echocardiographic measurement, BNP/NTproBNP, and clinical notes. Patients with incomplete datasets were excluded from the study.

The data on TSLV leads were compared to the data collected from patients who underwent a conventional trans-venous CRT upgrade via the CS.

### 2.2. Statistical Analysis

The statistical analysis was performed using Microsoft Excel (Microsoft Corp., Redmond, WA, USA), with the XLSTAT add-on (Addinsoft SARL, Paris, France). The descriptive analysis of the study group was performed with Excel, and normality tests (Anderson–Darling) and complex statistical tests (chi-squared, Wilcoxon, Frederick, MD, USA) were performed using XLSTAT. *p* < 0.05 was statistically significant.

### 2.3. Schematic Presentation of the Procedure

Seven cardiac resynchronization therapy defibrillators (CRTDs) and four cardiac resynchronization therapy pacemakers (CRTPs) were used. All 11 patients had active fixation right ventricular (RV) and right atrial (RA) leads implanted by the standard approach before the TSLV lead implant.

The procedure was performed under local anesthetic and mild sedation in eight cases and under general anesthesia in three. Both groins and the left pectoral area were prepared. Venous access was gained via the extra thoracic subclavian vein in four cases and subclavian access in seven cases. Ultrasound (US)-guided right femoral access was successfully gained in all 11 cases.

### 2.4. Trans-Septal Puncture

SL1 and Agilis (St. Jude Medical, St. Paul, MN, USA) sheaths were advanced via the RF. The trans-septal puncture was performed with a Brockenbrough needle (St. Jude Medical, St. Paul, MN, USA). The guidewire was advanced in the left upper pulmonary vein (LUPV) and the Agilis and SL1 were guided to the left atrium through the same aperture. Balloon inflation across the interatrial septum was performed next to the Agilis to create a large aperture to allow passage to the LV lead.

### 2.5. Trans-Septal LV Lead Placement

A pre-pectoral pocket was fashioned, venous access was gained, and active fixation RV leads were advanced to the right atrium. A GooseNeck snare (Medtronic, Minnesota, MN, USA) was passed through the multipurpose angio sheath (MPA) to the right atrium, under fluoroscopy guidance. The LV lead tip was grabbed by the GooseNeck snare. The stylet was pulled back from within the RV pacing lead, leaving it flexible, steered to the left atrium and through the mitral valve, and guided to the left ventricle. The GooseNeck snare was released, and the stylet was advanced within the pacing lead, which allowed for the active fixation of the lead endocardially.

Pacing thresholds and stability tests were performed, and the sheaths were removed. The lead was secured in the pre-pectoral pocket and attached to a CRT device.

**Figure 1:**
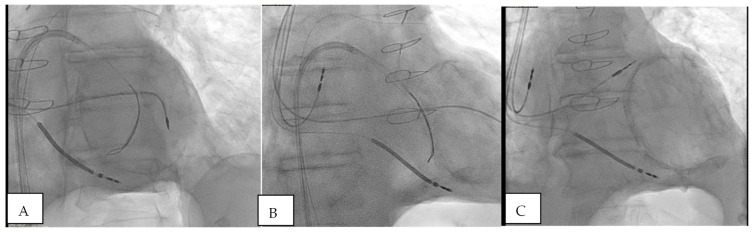
Fluoroscopic image of the GooseNeck snare and endocardial active fixation in a patient with prior extensive apical aneurysmectomy. Apical segments were silent electrically (**A**,**B**) and excellent electrical parameters were observed in a basal position (**C**).

Ten leads were positioned in the apical segments. In one case, due to a large apical segment aneurysm and previous extensive aneurysmectomy, the apical segments were silent electrically and the lead was fixed in a basal position with excellent electric parameters (Figure 1).

## 3. Results

### 3.1. Analysis of Trans-Septal Endocardial LV Lead

A total of 11 patients were successfully implanted with a TSLV. The sex ratio was 81% in favor of male patients (nine male and two female). The median age was 76 ± 9 years. Seven patients (63.64%) had >40% right ventricular pacing (RVP) and four (36.36%) patients had <40% RVP. Post-upgrade BiV pacing was >90% in all of the participants.

The mean pre-TSLV implant QRS duration was 170 ± 20 ms, compared to 114 ± 18 ms after upgrade, showing a narrower QRS duration of at least 57 ± 11 ms, which was a statistically significant difference with a *p*-value of <0.0038. The pre-TSLV implant LVESV was 160 ± 50 mL. After implantation, it was 131 ± 64 mL, with a post-implant decrease in LVESV of 29.73 ± 40 mL; The *p*-value of 0.0459 showed a statistically significant difference. The pre-implant LVIDd measured in 2M-mode was 6.15 ± 0.5 cm compared to 5.64 ± 0.8 cm after, showing a decrease of 0.51 ± 0.45 cm with a *p*-value of 0.0095. The pre-implant mean LVEDV was 224 ± 63 mL, and the post-implant LVEDV was 185 ± 85 mL, with a median decrease of 38.91 ± 58.46 mL and a *p*-value of 0.0453 showing a statistically significant difference.

The mean LVEF was 20 ± 9% before TSLV and 32 ± 15% after implantation, showing an increase of 11.91 ± 14.63%, which was statistically significant with a *p*-value of 0.0119.

The mean NYHA class before the implant was 3.6 ± 0.5 compared to 2.18 ± 0.6 post-implant, showing at least a one-grade classification decrease in the NYHA class, which was statistically significant with a *p*-value of 0.0049.

The pre-implant mean NTproBNP level was 2799 ± 2961 compared to a post-implant level of 2068 ± 2160. The median reduction of −731.45 ± 1638 was statistically significant with a *p*-value of 0.0082.

Five (45.45%) patients were on direct oral anticoagulants (DOACs) and six (53.55%) were on warfarin with a target INR of 2.5–3.5. Only 1 of the 11 patients (9.09%) was admitted with a thromboembolic CVA; this patient was anticoagulated with direct oral anticoagulants (DOAC). None of the patients on Warfarin had a documented CVA following TSLV implantation. This thromboembolic event happened 3 months after the implantation and the patient fully recovered. The patient pacing checks showed no evidence of atrial arrhythmia to explain the CVA and the carotid Doppler showed no disease; hence, it was attributed to the TSLV lead and the DOAC was switched to warfarin with a target INR of 2.5–3.5. No further embolic events were reported.

No post-TSLV infections or pneumothorax were reported. One case of post-procedural pericardial effusion was treated conservatively. SVC-related stenosis was reported in four cases, all at the level of the axillary and subclavian veins, but the operators could guide a terumo wire past the stenosis.

The distribution of patients based on the etiology of cardiomyopathy, medication history, and associated comorbidities for the TSLV group as well as the conventional upgrade to CRT is listed in Table 1.

### 3.2. Analysis of TSLV Compared to Trans-Venous CRT Upgrade

Since we demonstrated that TSLV implantation improves both clinical and echocardiographic parameters, we then compared these parameters with those who had a trans-venous CRT upgrade from either a conventional pacemaker to CRT-P or an ICD to a CRT-D. A total of 151 patients were included (93 CRT-P and 58 CRT-D). Patients’ clinical and echocardiographic parameters were analyzed to facilitate this comparative analysis; the descriptive analysis is detailed in Table 1.

Since this was a retrospective analysis, ensuring that the two groups were fairly comparable was critical to minimize bias and confounding variables. Patients who undergo an upgrade to CRT are typically a higher-risk cohort compared to those receiving de novo CRT implants. Hence, for the comparative analysis, we used upgrade to CRT rather than de novo implant as a comparison for trans-septal LV lead implantation for a more equitable assessment between the groups. This choice helps control for confounding factors that may skew results when comparing patients with de novo CRT implant, who typically have fewer complications and different baseline characteristics.

**Figure 2:**
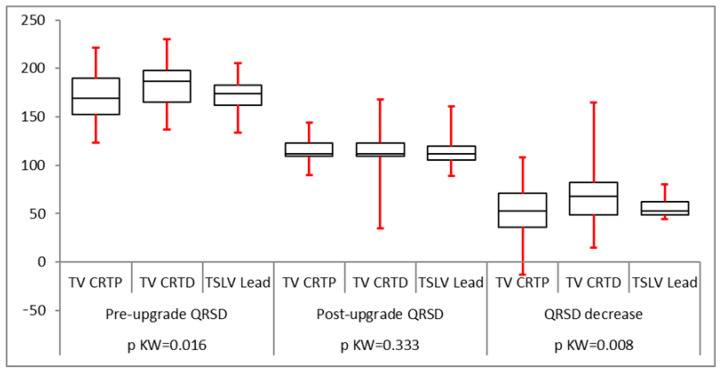
QRS duration was significantly narrower in all three groups following an LV lead implant.

**Figure 3:**
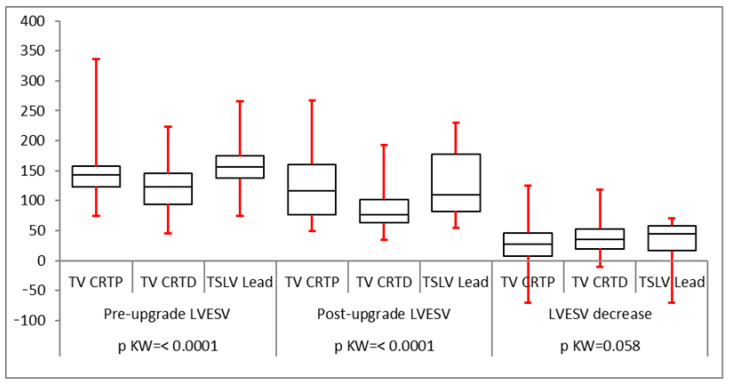
Reduction in LVESV after LV lead implantation was statistically significant in all three groups.

**Figure 4:**
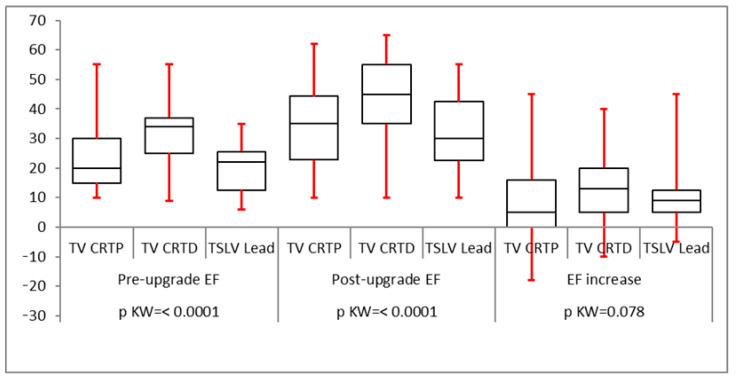
There was a significant improvement in LVEF following an LV lead in all three subgroups, which was statistically significant. No subgroup was superior to the other subgroups.

**Figure 5:**
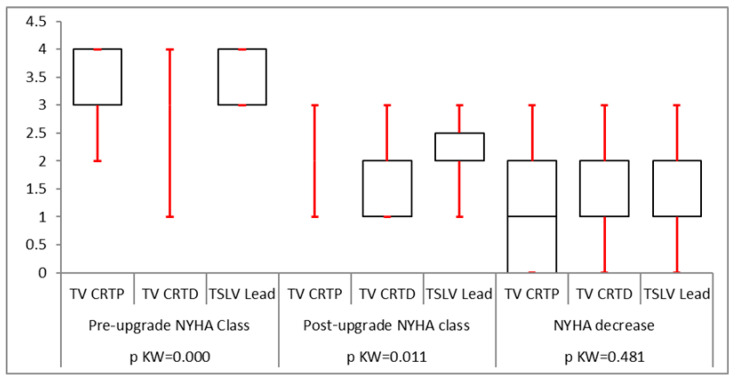
There was significant reduction in functional NYHA class following an LV lead in all three subgroups, which was statistically significant. No subgroup was superior to the other subgroups.

All groups had a statistically significant reduction in QRS duration following LV lead implantation (Figure 2). The mean pre-implant LVESV was greater than for trans-venous CRT upgrades (160 ± 50 mL compared to 121 ± 33 mL in the CRT-P upgrade group and 151 ± 47 mL in the CRT-D upgrade group) which was statistically significant. However, post LV lead implantation, the reduction in LVESV was statistically similar in all groups with a *p*-value of 0.0584 (Figure 3). LVEDV was also greater in the TSLV group before implantation (224 ± 63 mL vs. 170 ± 50 mL in CRT-P upgrade patients and 219 ± 69 mL in CRT-D). The post-LV lead implantation reduction in LVEDV was statistically not different (*p* = 0.69), as illustrated in Table 2. The mean ejection fraction (EF) before TSLV implantation was statistically lower in the TSLV group compared to the trans-venous CRT upgrade group (20 ± 9% vs. 30.5 ± 9.7 in the CRT-P group and 23.88 ± 11 in the CRT-D group). The post-LV lead implant increase in EF was similar in all groups, as shown in Figure 4. Patients in the TSLV group had a greater NYHA class pre-TSLV implant (3.64 ± 0.5 vs. 2.88 ± 0.55 in the CRT-P group and 3.10 ± 0.7 in the CRT-D group) which was significant. However, the reduction in NYHA class was not statistically significant between the groups, as illustrated in Figure 5.

**Figure 6:**
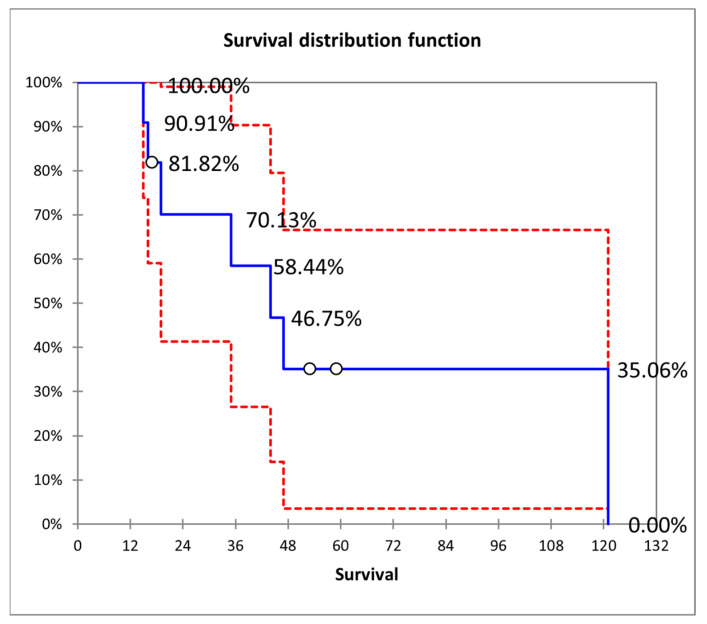
Survival rate post TSLV implantation.

The overall survival rate post implantation was 100% within the first 12 months, 66.67% at 24 months, 55.55% at 36 months, 33% at 48 months, and 14.29% at 60 months, as illustrated in Figure 6.

## 4. Discussion

This study demonstrated that implanting a TSLV lead was safe and it improved EF, LVEDV, LVESV, and LVIDd. It also reduced NTproBNP levels. Functionally, patients were in a better NYHA class following TSLV lead implantation.

Studies have analyzed the safety and long-term effects of TSLV and other techniques used to deliver an LV lead trans-septally into the left ventricle. Morino Vasquez et al. demonstrated that the TSLV approach was safe and a better alternative than the surgical epicardial LV lead [20]. In a comparative analysis of ECG markers of repolarization after trans-septal, CS, and surgical epicardial LV leads, Scot et al. illustrated that the endocardial LV lead implant was associated with repolarization characteristics that were less arrhythmogenic compared to epicardial LV leads [21].

The snare coupling technique, reported in a case series involving five patients by Patel et al., is a simple and reliable approach to implanting an endocardial LV lead [22]. The GooseNeck snare technique used in this study seems to be reliable, with a high success rate and no high risk of complications. A high incidence of stroke among these patients has remained a major problem, making the procedure less attractive. In a study by Geller et al., LVEF increased by a median of 27%, and LVEDD and LVESD also showed statistically significant improvements at 3 months. However, the rate of stroke contribuiting to trans-septal LV lead implant was reported to be approximately 7% [23]. In another large study by Gamble et al., involving 384 patients from 23 studies, the rate of thromboembolic complications was reported by all studies over 22 ± 32 months, with a stroke rate of 2.5 events per 100 patients and 2.6 transient ischemic attacks per 100 patients [24].

In our study, the incidence of stroke was approximately 9%. This might represent a small number anomaly, but we observed that appropriate anticoagulation with warfarin was not associated with the risk of systemic embolization.

Patients within this group who underwent a TSLV lead implant due to a failed trans-venous approach had more advanced heart failure, defined by their higher NYHA class compared to conventional trans-venous CRT implant groups. Their pre- implant, echocardiographic parameters were also worse compared to the trans-venous CRT implant group. These patients maybe at a high risk for a surgical approach to implant an LV lead, due to their significant heart failure and poor echocardiographic parameters. Studies have been conflicting regarding safety and overall survival following a surgical epicardial LV lead implant. Studies have demonstrated the overall safety and effectiveness of a surgically placed epicardial LV lead [25,26,27]. However, in a study by Miller et al., the mortality rate amongst patients undergoing surgical LV implants was higher compared to trans-venous CRT implants [28].

With the emergence of conduction system pacing and the rapid development of left bundle area pacing as a safe alternative to CRT, alongside the leadless left ventricular stimulation with WiSE-CRT, we might be taking a step away from trans-septal LV endocardial lead implantation. We may instead choose conduction system pacing or leadless left ventricular stimulation with WiSE-CRT in patients with a failed CS approach as the first alternative.

Although, studies have been promising, randomized controlled trials will be necessary to provide more evidence on the safety and reliability of conduction system pacing and leadless LV stimulation.

In a large observational study, involving 632 patients with attempted LBBAP, Su et al. demonstrated that the procedure was successful in 97.8% of patients and was associated with improvement in EF in patients with impaired LV systolic function and QRS > 120 ms [29].

In phase I of the SOLVE-CRT study, Okabe et al. reported a high success rate of LV endocardial electrode placement and favorable echocardiographic changes in EF, LVEDV, and LVESV after WiSE-CRT. Hower, device-related complications were reported in 9.7% of the patients such as insufficient LV pacing, the embolization of an un-anchored LV electrode, and wound infection [16].

In the left bundle branch area pacing population, in a study by Jastrzebski et al., intraprocedural perforation into the LV was reported in 3.67% of patients [30].

Poor CS anatomy and septal scarring with irreversible left bundle branch block (LBBB) give rise to non-response to CRT [31,32]. The same applies to conduction system pacing. A study by Strocchi et al. demonstrated that severe LV HIS-Purkinje conduction system disease and septal scars make CSP ineffective. Interestingly, they showed that selective-LBBAP caused dyssynchrony due to delayed RV activation, compared to RV preexcitation. Their study also found that although left ventricle activation time (LVAT) was reduced with LBBAP, interventricular dyssynchrony was not [33].

TSLV lead overcomes these issues in selected patients. This highlights the need for personalized approaches in heart failure management, ensuring that patients receive the most appropriate therapy based on their unique anatomical and clinical profiles. Trans-septal LV endocardial lead is also capable of more effective electromechanical resynchronization due to a wider choice of pacing sites [34].

This study demonstrates that alongside conduction system pacing, leadless endocardial lead placement, and surgical LV lead placement, TSLV remains another alternative in cases where trans-venous CRT implantation fails. This might build a case for ongoing trials comparing various alternatives to conventional CRT and identifying better options for this group of patients.

## 5. Conclusions

Trans-septal endocardial LV lead implantation in patients with a failed CS approach remains an alternative. However, a higher incidence of stroke exists among these patients, as well as a greater procedure-related risk of complications compared to conventional CRT.

Implanting a TSLV lead has a high success rate. It improves EF, LVEDV, LVESV, and LVIDd. It also significantly reduces NTproBNP levels and NYHA class compared to de novo implants via CS.

A multidisciplinary approach and patient selection are very important in this group of patients.

Limitations:

This is a retrospective study with all the limitations of a non-randomized study. The post-TSLV systemic embolic was approximately 9%; this might represent a small anomaly effect and not reflect the true incidence rate of systemic embolization after TSLV implantation. Follow-up investigations of our patients varied, not providing the correct conditions for comparison between the groups. We used CRT upgrade as a comparison for trans-septal LV lead to make the comparison fairer. Moreover, the survival data were available only for the TSLV group. Potential future prospective studies can help illustrate differences in survival rates and other relevant endpoints and provide more robust evidence to support the findings.

## Figures and Tables

**Table 1:** Descriptive analysis of TSLV lead and conventional CRT upgrade.

	All Patients	Patients with Conventional Upgrade to CRT	TSLV Lead	*p* Chi Square
Age	79 ± 9 (162)	82 ± 10 (151)	76 ± 9 (11)	0.317
Sex				0.412
Female	47 (25%)	45 (31%)	2 (19%)	
Male	115 (75%)	106 (69%)	9 (81%)	
Rhythm				0.789
Sinus Rhythm	112 (70%)	104 (69%)	8 (72.7%)	
Atrial Arrythmia	50 (30%)	47 (31%)	3 (27.3%)	
Etiology				
IHD	85 (61%)	77 (50%)	8 (72.7%)	0.163
Non-IHD	80 (43%)	77 (40%)	3 (27.7%)	0.605
ICCs	15 (11.5%)	14 (10.38%)	1 (9%)	0.984
VHD	19 (14%)	17 (10%)	2 (18%)	0.491
HF Medications				
Beta-Blockers	162 (100%)	151 (100%)	11 (100%)	0.43
MRAs	133 (85%)	123 (80%)	10 (90.9%)	0.43
ARNi	89 (59%)	82 (55%)	7 (63.4%)	0.548
SGLT-2	84 (54.5%)	77 (51.5%)	7 (63.4%)	0.394
Comorbidities				
Diabetes	67 (45.5%)	62 (24.7%)	5 (45.4%)	0.775
Hypertension	143 (92.5%)	132 (85%)	11 (100%)	0.21
CKD	67 (45.5%)	65 (42.5%)	2 (27.7%)	0.44
	57 (35.7%)	53 (34.7%)	4 (36.36%)	
	14 (13.5%)	12 (8.5%)	2 (18.18%)	
	14 (13.5)	12 (8.5%)	2 (18.18%)	

IHD: Ischemic Heart Disease; non-IHD: Non-ischemic Heart Disease; ICCs: Inherited Cardiac Conditions; VHD: Valvular Heart Disease; HF: Heart Failure; MRAs: Mineralocorticoid Receptor Antagonists; ARNi: Angiotensin Receptor-Neprilysin Inhibitor; SGLT-2: Sodium-Glucose Transport Protein 2 Inhibitors.

**Table 2:** Statistical analysis of echocardiographic parameters, NTproBNP, and NYHA functional class pre- and post-cardiac resynchronization therapy, comparing trans-venous and trans-septal approaches.

	Median (IQR)	NTproBNP	LVESV	LVEDV	LVIDd
Pre-upgrade	TV CRTP		142.5 (123.5–157)	209 (175.5–262.75)	6.2 (5.8–6.6)
	TV CRTD		123 (94–145)	160 (131–195)	5.6 (5.2–6)
	TSLV Lead	1606 (1059–3098.5)	156 (137–174.5)	210 (189–251.5)	6 (5.75–6.55)
Post-upgrade	TV CRTP		117 (76.5–160.75)	157 (127.75–221)	5.95 (5–6.575)
	TV CRTD		77 (63–102)	112 (98–152)	5 (4.7–5.7)
	TSLV Lead	1058 (557.5–2552.5)	110 (82–178)	176 (125.5–238)	5.4 (4.95–6.25)
Reduction	TV CRTP		27 (8–46)	32.5 (18.25–59)	0.3 (0–0.9)
	TV CRTD		35 (20–53)	42 (23–53)	0.6 (0.3–0.7)
	TSLV Lead	−412 (−958.5–168)	45 (16.5–58)	43 (34.5–55.5)	0.5 (0.3–0.8)

TV CRTP = Trans-venous cardiac resynchronization therapy-pacemaker; TV CRTD = Trans-venous cardiac resynchronization therapy-defibrillator; TSLV = Trans-septal left ventricle; LVIDd = Left ventricle internal diameter in diastole; IQR = Interquartile range; LVESV = Left ventricle end-systolic volume; LVEDV = Left ventricle end-diastolic volume.

## Data Availability

The data presented in this study are available on request from the corresponding author. The data are not publicly available due to patients’ privacy rights.

## References

[1] Linde C., Abraham W.T., Gold M.R., St John Sutton M., Ghio S., Daubert C., REVERSE (REsynchronization reVErses Remodeling in Systolic left vEntricular dysfunction) Study Group (2008). Randomized trial of cardiac resynchronization in mildly symptomatic heart failure patients and in asymptomatic patients with left ventricular dysfunction and previous heart failure symptoms. J. Am. Coll. Cardiol..

[2] Anand I.S., Carson P., Galle E., Song R., Boehmer J., Ghali J.K., Jaski B., Lindenfeld J., O’Connor C., Steinberg J.S. (2009). Cardiac resynchronization therapy reduces the risk of hospitalizations in patients with advanced heart failure: Results from the Comparison of Medical Therapy, Pacing and Defibrillation in Heart Failure (COMPANION) trial. Circulation.

[3] Kutyifa V., Kloppe A., Zareba W., Solomon S.D., McNitt S., Polonsky S., Barsheshet A., Merkely B., Lemke B., Nagy V.K. (2013). The influence of left ventricular ejection fraction on the effectiveness of cardiac resynchronization therapy: MADIT-CRT (Multicenter Automatic Defibrillator Implantation Trial With Cardiac Resynchronization Therapy). J. Am. Coll. Cardiol..

[4] Cleland J.G., Daubert J.C., Erdmann E., Freemantle N., Gras D., Kappenberger L., Klein W., Tavazzi L., CARE-HF study Steering Committee and Investigators (2001). The CARE-HF study (CArdiac REsynchronisation in Heart Failure study): Rationale, design and end-points. Eur. J. Heart Fail..

[5] Curtis A.B., Worley S.J., Chung E.S., Li P., Christman S.A., St John Sutton M. (2016). Improvement in Clinical Outcomes With Biventricular Versus Right Ventricular Pacing: The BLOCK HF Study. J. Am. Coll. Cardiol..

[6] Merkely B., Kosztin A., Roka A., Geller L., Zima E., Kovacs A., Boros A.M., Klein H., Wranicz J.K., Hindricks G. (2017). Rationale and design of the BUDAPEST-CRT Upgrade Study: A prospective, randomized, multicentre clinical trial. Europace.

[7] Pujol-Lopez M., Jiménez-Arjona R., Garre P., Guasch E., Borràs R., Doltra A., Ferró E., García-Ribas C., Niebla M., Carro E. (2022). Conduction System Pacing vs Biventricular Pacing in Heart Failure and Wide QRS Patients: LEVEL-AT Trial. JACC Clin. Electrophysiol..

[8] Whinnett Z.I., Shun-Shin M.J., Tanner M., Foley P., Chandrasekaran B., Moore P., Adhya S., Qureshi N., Muthumala A., Lane R. (2023). Effects of haemodynamically atrio-ventricular optimized His bundle pacing on heart failure symptoms and exercise capacity: The His Optimized Pacing Evaluated for Heart Failure (HOPE-HF) randomized, double-blind, cross-over trial. Eur. J. Heart Fail..

[9] León A.R., Abraham W.T., Curtis A.B., Daubert J.P., Fisher W.G., Gurley J., Hayes D.L., Lieberman R., Petersen-Stejskal S., Wheelan K. (2005). Safety of transvenous cardiac resynchronization system implantation in patients with chronic heart failure: Combined results of over 2000 patients from a multicenter study program. J. Am. Coll. Cardiol..

[10] Gamble J.H.P., Herring N., Ginks M., Rajappan K., Bashir Y., Betts T.R. (2016). Procedural success of left ventricular lead placement for cardiac resynchronization therapy: A meta-analysis. JACC Clin. Electrophysiol..

[11] Ypenburg C., Schalij M.J., Bleeker G.B., Steendijk P., Boersma E., Dibbets-Schneider P., Stokkel M.P., van der Wall E.E., Bax J.J. (2007). Impact of viability and scar tissue on response to cardiac resynchronization therapy in ischaemic heart failure patients. Eur. Heart J..

[12] Gellér L., Salló Z., Molnár L., Tahin T., Özcan E.E., Kutyifa V., Osztheimer I., Szilágyi S., Szegedi N., Ábrahám P. (2019). Long-term single-centre large volume experience with transseptal endocardial left ventricular lead implantation. Europace.

[13] van Gelder B.M., Scheffer M.G., Meijer A., Bracke F.A. (2007). Transseptal endocardial left ventricular pacing: An alternative technique for coronary sinus lead placement in cardiac resynchronization therapy. Heart Rhythm..

[14] Neuhoff I., Szilágyi S., Molnár L., Osztheimer I., Zima E., Dan G.A., Merkely B., Gellér L. (2016). Transseptal Leftventricular Endocardial Pacing is an Alternative Technique in Cardiac Resynchronization Therapy. One year experience in a high volume center. Rom. J. Intern. Med..

[15] Wijesuriya N., Elliott M.K., Mehta V., Sidhu B.S., Strocchi M., Behar J.M., Niederer S., Rinaldi C.A. (2022). Leadless Left Bundle Branch Area Pacing in Cardiac Resynchronisation Therapy: Advances, Challenges and Future Directions. Front. Physiol..

[16] Okabe T., Hummel J.D., Bank A.J., Niazi I.K., McGrew F.A., Kindsvater S., Oza S.R., Scherschel J.A., Walsh M.N., Singh J.P. (2022). Leadless left ventricular stimulation with WiSE-CRT System—Initial experience and results from phase I of SOLVE-CRT Study (nonrandomized, roll-in phase). Heart Rhythm..

[17] Chung M.K., Patton K.K., Lau C.P., Dal Forno A.R.J., Al-Khatib S.M., Arora V., Birgersdotter-Green U.M., Cha Y.M., Chung E.H., Cronin E.M. (2023). 2023 HRS/APHRS/LAHRS guideline on cardiac physiologic pacing for the avoidance and mitigation of heart failure. Heart Rhythm..

[18] Shen J., Jiang L., Wu H., Li H., Zhong J., Pan L. (2022). Case report: Left bundle branch pacing guided by real-time monitoring of current of injury and electrocardiography. Front. Cardiovasc. Med..

[19] Morgan J.M., Biffi M., Gellér L., Leclercq C., Ruffa F., Tung S., Defaye P., Yang Z., Gerritse B., Van Ginneken M. (2016). ALternate Site Cardiac ResYNChronization (ALSYNC): A prospective and 355 multicentre study of left ventricular endocardial pacing for cardiac resynchronization therapy. Eur. Heart J..

[20] Moriña-Vázquez P., Roa-Garrido J., Fernández-Gómez J.M., Venegas-Gamero J., Pichardo R.B., Carranza M.H. (2013). Direct left ventricular endocardial pacing: An alternative when traditional resynchronization via coronary sinus is not feasible or effective. PACE-Pacing Clin. Electrophysiol..

[21] Scott P.A., Yue A.M., Watts E., Zeb M., Roberts P.R., Morgan J.M. (2011). Transseptal left ventricular endocardial pacing reduces dispersion of ventricular repolarization. PACE-Pacing Clin. Electrophysiol..

[22] Patel M.B., Worley S.J. (2013). Snare coupling of the pre-pectoral pacing lead delivery catheter to the femoral transseptal apparatus for endocardial cardiac resynchronization therapy: Mid-term results. J. Interv. Card. Electrophysiol..

[23] Geller L., Merkely B., Molnar L., Szilagyi S.Z., Zima E., Szeplaki G., Osztheimer I., Tahin T., Ozcan E.E., Apor A. (2014). Long term efficacy and safety of transseptal endocardial left ventricular lead implantation after left ventricular lead implantations. Eur. Heart J..

[24] Gamble J.H., Herring N., Ginks M., Rajappan K., Bashir Y., Betts T.R. (2018). Endocardial left ventricular pacing for cardiac resynchronization: Systematic review and meta-analysis. Europace.

[25] Kim H.R., Lim K., Park S.J., Park J.S., Kim J.Y., Chung S., Jung D.S., Park K.M., On Y.K., Kim J.S. (2022). Thoracoscopic Implantation of Epicardial Left Ventricular Lead for Cardiac Resynchronization Therapy. J. Cardiovasc. Dev. Dis..

[26] Navia J.L., Atik F.A., Grimm R.A., Garcia M., Vega P.R., Myhre U., Starling R.C., Wilkoff B.L., Martin D., Houghtaling P.L. (2005). Minimally invasive left ventricular epicardial lead placement: Surgical techniques for heart failure resynchronization therapy. Ann. Thorac. Surg..

[27] Nelson K.E., Bates M.G., Turley A.J., Linker N.J., Owens W.A. (2013). Video-assisted thoracoscopic left ventricular pacing in patients with and without previous sternotomy. Ann. Thorac. Surg..

[28] Miller A.L., Kramer D.B., Lewis E.F., Koplan B., Epstein L.M., Tedrow U. (2011). Event-free survival following CRT with surgically implanted LV leads versus standard transvenous approach. Pacing Clin. Electrophysiol..

[29] Su L., Wang S., Wu S., Xu L., Huang Z., Chen X., Zheng R., Jiang L., Ellenbogen K.A., Whinnett Z.I. (2021). Long-Term Safety and Feasibility of Left Bundle Branch Pacing in a Large Single-Center Study. Circ. Arrhythm. Electrophysiol..

[30] Jastrzębski M., Kiełbasa G., Cano O., Curila K., Heckman L., De Pooter J., Chovanec M., Rademakers L., Huybrechts W., Grieco D. (2022). Left bundle branch area pacing outcomes: The multicentre European MELOS study. Eur. Heart J..

[31] Sieniewicz B.J., Gould J., Porter B., Sidhu B.S., Teall T., Webb J., Carr-White G., Rinaldi C.A. (2019). Understanding non-response to cardiac resynchronisation therapy: Common problems and potential solutions. Heart Fail. Rev..

[32] Larsen C.K., Duchenne J., Galli E., Aalen J.M., Bogaert J., Lederlin M., Kongsgaard E., Linde C., Penicka M., Donal E. (2021). Septal scar predicts non-response to cardiac resynchronization therapy. Eur. Heart J. Cardiovasc. Imaging.

[33] Strocchi M., Gillette K., Neic A., Elliott M.K., Wijesuriya N., Mehta V., Vigmond E.J., Plank G., Rinaldi C.A., Niederer S.A. (2023). Effect of scar and His-Purkinje and myocardium conduction on response to conduction system pacing. J. Cardiovasc. Electrophysiol..

[34] Elliott M.K., Mehta V.S., Sidhu B.S., Niederer S., Rinaldi C.A. (2021). Endocardial left ventricular pacing. Herz.

